# Palmprint Recognition across Different Devices

**DOI:** 10.3390/s120607938

**Published:** 2012-06-08

**Authors:** Wei Jia, Rong-Xiang Hu, Jie Gui, Yang Zhao, Xiao-Ming Ren

**Affiliations:** 1 Institute of Nuclear Energy Safety Technology, Chinese Academy of Science, Hefei 230031, China; E-Mail: icg.jiawei@gmail.com; 2 Institute of Intelligent Machines, Chinese Academy of Science, Hefei 230031, China; E-Mails: guijiejie@gmail.com (J.G.); zyknight@mail.ustc.edu.cn (Y.Z.); rxm1989.happy@gmail.com (X.-M.R.); 3 Department of Automation, University of Science and Technology of China, Hefei 230027, China

**Keywords:** biometrics, palmprint recognition, different devices, sensors

## Abstract

In this paper, the problem of Palmprint Recognition Across Different Devices (PRADD) is investigated, which has not been well studied so far. Since there is no publicly available PRADD image database, we created a non-contact PRADD image database containing 12,000 grayscale captured from 100 subjects using three devices, *i.e.*, one digital camera and two smart-phones. Due to the non-contact image acquisition used, rotation and scale changes between different images captured from a same palm are inevitable. We propose a robust method to calculate the palm width, which can be effectively used for scale normalization of palmprints. On this PRADD image database, we evaluate the recognition performance of three different methods, *i.e.*, subspace learning method, correlation method, and orientation coding based method, respectively. Experiments results show that orientation coding based methods achieved promising recognition performance for PRADD.

## 1. Introduction

In recent years, palmprint recognition has drawn widespread attention from researchers. Generally, palmprint recognition involves using the person's palm to identify who the person is or verify whether the person is “whom he claims to be”. Some previous researches have shown that, compared with fingerprints or iris- based personal biometrics systems, palmprint-based biometric systems have several special advantages such as rich features, less distortion and easy self-positioning [1–6]. And, it can also obtain high accurate recognition rate with fast processing speed [2–6]. For the aforementioned reasons, nowadays research on palmprint recognition is becoming more and more active [5,6].

Roughly speaking, the techniques of palmprint recognition can be divided into two categories, *i.e.*, 2-D based [5] and 3-D based [7], respectively. As their name suggests, 2-D based palmprint recognition techniques capture a 2-D image of the palm surface and use it for feature extraction and matching, while 3-D based techniques capture the 3-D depth information for recognition. As noted in the literature [7], 3-D palmprint recognition techniques offer some special advantages. For example, they are robust to illumination variations, contaminations and spoof attacks. However, the cost of 3-D data acquisition devices is high, which limits the usage of 3-D palmprint recognition techniques [7]. Therefore, 2-D palmprint recognition has drawn more attention in the past decade [6]. In this paper, we also focus on it.

It is well known that the palm contains rich features such as minutiae, ridges, principal lines and creases. In a high-resolution (500 ppi or higher) palmprint image, all features mentioned above can be extracted. Recently, there have been several works related to high-resolution palmprint recognition [8,9]. In fact, most high-resolution palmprint recognition techniques are mainly developed for forensic applications as about 30 percent of the latents recovered from crime scenes are from palms [9]. On the other hand, for civil applications, the technique of low-resolution (about 100 ppi) palmprint recognition is enough for robust personal authentication. In this paper, our work also belongs to the low-resolution palmprint recognition category. In a low-resolution palmprint image, only principal lines and creases can be extracted to construct features. In the early stages of the study for low-resolution palmprint recognition, the inked offline methods were investigated [10]. However, the quality of inked palmprint image is very poor, therefore, researchers' interest later turned to online palmprint recognition. Zhang *et al.* proposed the first online low-resolution palmprint recognition system, and published a palmprint image database, *i.e.*, the PolyU database [5]. After that, research on palmprint recognition grew rapidly. In order to acquire low-resolution palmprint images, different devices were exploited. Ribaric *et al.* [11] used a digital scanner to collect palmprint images. Zhang *et al.* [5] and Sun *et al.* [12] developed CCD camera-based special devices for palmprint acquisition, respectively. Kumar *et al.* captured hand images using a digital camera [13]. In their works [5,11–13], the palmprint images were captured in the contact manner. Recently, there are more studies on contact-free palmprint recognition. Usually, web-cameras [14], cameras in smart phones, panel PCs, or notebook PCs were used to collect contact-free palmprint images.

So far, many approaches have been proposed for low-resolution palmprint recognition. Kong *et al.* [6] made a survey of these approaches and divided them into several different categories such as texture based, palm line based, subspace learning based, orientation coding based, correlation based, local image descriptor based, and multi-feature based, respectively. From the literature [6], it can be seen that most research works have focused on feature extraction and matching. In order to improve the recognition performance, other strategies were exploited. For example, Zhang *et al.* [15] proposed multi-spectral based palmprint recognition. Here, it should be noted that all of the previous studies of palmprint recognition only used one device to collect palmprint images. That is, the training set and test set were captured using a same device.

In this paper, we investigate the problem of Palmprint Recognition Across Different Devices (PRADD), which has not been well studied so far. In fingerprint-based biometrics, the problem of biometric sensor interoperability has been investigated [16–18]. Biometric sensor interoperability refers to the ability of a system to compensate for the variability introduced in the biometric data of an individual due to the deployment of different sensors [18]. From the literature [16–18], it can be seen that poor inter-sensor performance has been reported for fingerprint recognition.

With the wide applications of palmprint recognition and the popularization of all kinds of cameras, there is a high possibility that a person's palmprint images would be captured by different devices. Therefore, the problem of PRADD needs to be carefully studied. The technique of PRADD has the following potential applications: (1) Remote enrollment in a palmprint based distributed biometrics system. For example, when a user plans to attend a meeting which will be held in another city far away, at first he may be required to provide his palmprint images captured by his camera. In this way, this user's identity can be directly checked by another device at the meeting site. (2) Personal authentication anywhere. For example, if one person's palmprint images have been recorded by a digital camera in the police station, the police can search for this person anywhere using other devices such as smart-phones with cameras. Consequently, the PRADD technique is very useful to look for a lost elderly person or a suspect. (3) Palmprint based biometrics in cloud computing. In the cloud computing environment, palmprint based biometrics can become a service in which the technique of PRADD is needed. For example, palmprint recognition can be a service of the cloud computing for personal authentication on a smart-phone. A user can register palmprints using his old smart-phone. When he buys a new smart-phone, he does need to not register the palmprints again. Also, the registered palmprints captured by the smart-phone can be used for personal authentication in a user's other consumer electronics products such as a panel PC, or a notebook PC.

In order to study the PRADD technique, we create a non-contact palmprint image database using three devices, *i.e.*, one digital camera and two smart-phones. With the widespread application of digital cameras and smart-phones, the PRADD technique will be mainly used in such consumer electronics products, thus they were used to capture palmprint images in this work.

The main contributions of our work are as follows: first, it is the first time the problem of PRADD is investigated, which enriches the research on palmprint recognition. Second, a robust method to calculate the palm width is proposed, which can be effectively used for scale normalization of palmprints. Third, we evaluate the recognition performance for PRADD of three different methods, *i.e.*, subspace learning method, correlation method and orientation coding based method, respectively. Lastly, we create the first PRADD image database.

The rest of this paper is organized as follows: Section 2 describes the image collection and the preprocessing algorithm. Section 3 provides a brief review of some recognition methods. Section 4 reports the experimental results, and Section 5 concludes the whole paper.

## 2. Palmprint Image Collection and Preprocessing

**Figure 1.**
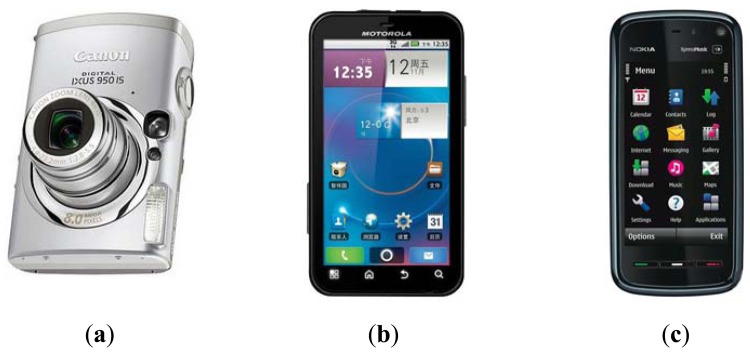
Three devices used for PRADD palmprint image collection: (**a**) the Canon IXUS 950 IS (C950) digital camera; (**b**) the Motorola ME525 (M525) smart-phone; (**c**) the Nokia 5800 XpressMusic (N5800) smart-phone.

In this paper, three popular consumer electronics products including one digital camera and two smart-phones were used to collect palmprint images. As shown in Figure 1, they are the Canon IXUS 950 IS (C950) digital camera, and the Motorola ME525 (M525) and Nokia 5800 XpressMusic (N5800) smart-phones, respectively. The C950 captures images using a CCD sensor with 800 million pixels while the M525 and N5800 capture images using CMOS sensors with 500 and 300 million pixels, respectively.

**Figure 2.**
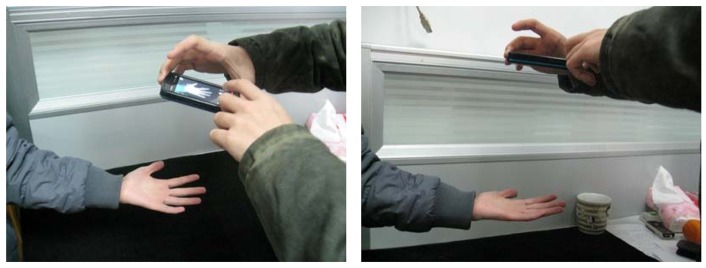
The scenes of non-contact palmprint image acquisition in this work.

The scenes of non-contact image acquisition are illustrated in Figure 2. During image acquisition, the hand with the fingers separated was placed above a table. In order to facilitate image segmentation, the table was covered by a black cloth. Meanwhile, the palmprint images were collected under indoor and daylight conditions.

**Figure 3.**
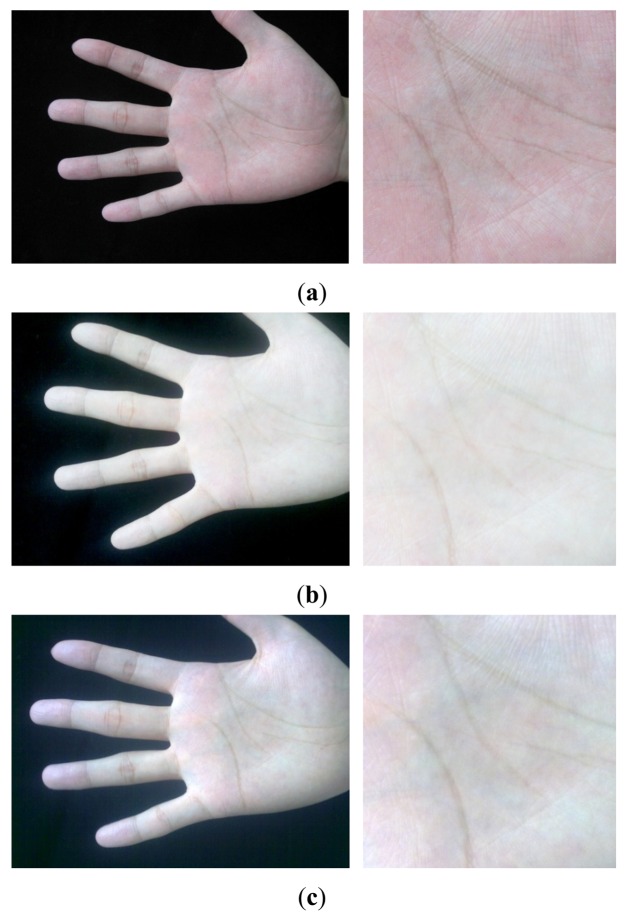
Three palmprint images and corresponding ROI images captured from a same palm by three devices: (**a**) the palmprint images and ROI images captured by the C950 camera; (**b**) the palmprint images and ROI images captured by the M525 smart-phone; (**c**) the palmprint images and ROI images captured by the N5800 smart-phone.

The sizes of raw images captured by the C950, M525 and N5800 are 3,264 × 2,448, 2,592 × 1,936, and 2,048 × 1,536 pixels, respectively, which are too large to be processed fast. Therefore, the raw images were resized into smaller ones, whose sizes are 816 × 612, 778 × 581, and 816 × 612 pixels, respectively. At the same time, we converted the images from color space to gray space. Figure 3 shows three palmprint images and their corresponding Regions of Interest (ROIs) captured by the three different devices from a same palm. It can be seen that the quality of images captured by the C950 is the best, and it seems that the quality of images captured by M525 is a little better than that of the N5800 according to our observations on the whole database.

Using the three devices introduced above, we created a PRADD image database named Chinese Academy of Science—HeFei Institutes of Physical Science (CASHF) image database. The CASHF database contains 12,000 grayscale palmprint images captured from 200 hands corresponding to 100 individuals. The volunteers are staff or students of the HeFei Institutes of Physical Science, and are all Chinese. Thirty one of them are female, and most of them are 22∼35 years old. During the image acquisition, there were no special requests concerning volunteers' rings and nails. That is, the volunteer can decide whether to wear a ring or trim the nails by him/herself.

Since three devices were used for data collection, the CASHF database consists of three sub-databases, named as N5800, M525 and C950, respectively, according to the names of the capturing devices. Each sub-database contains 20 samples captured from each of the hands in two sessions, where 10 samples were captured in the first session and the second session, respectively. That is, each device collected 4,000 palmprint images in total. Consequently, the total number of palmprint images captured by three devices is 12,000. Obviously, the total numbers of images captured in the first session and the second session are all 6,000 in this database. The average interval between the first and the second collection is about ten days. After image acquisition, the next task is to perform preprocessing. In our image acquisition, rotation and scale changes between different images captured from a same palm are inevitable, caused by the non-contact image acquisition. Thus, several tasks should be done in preprocessing stage, *i.e.*, rotation normalization, scale normalization, and ROI extraction.

### 2.1. Rotation Normalization

**Figure 4.**
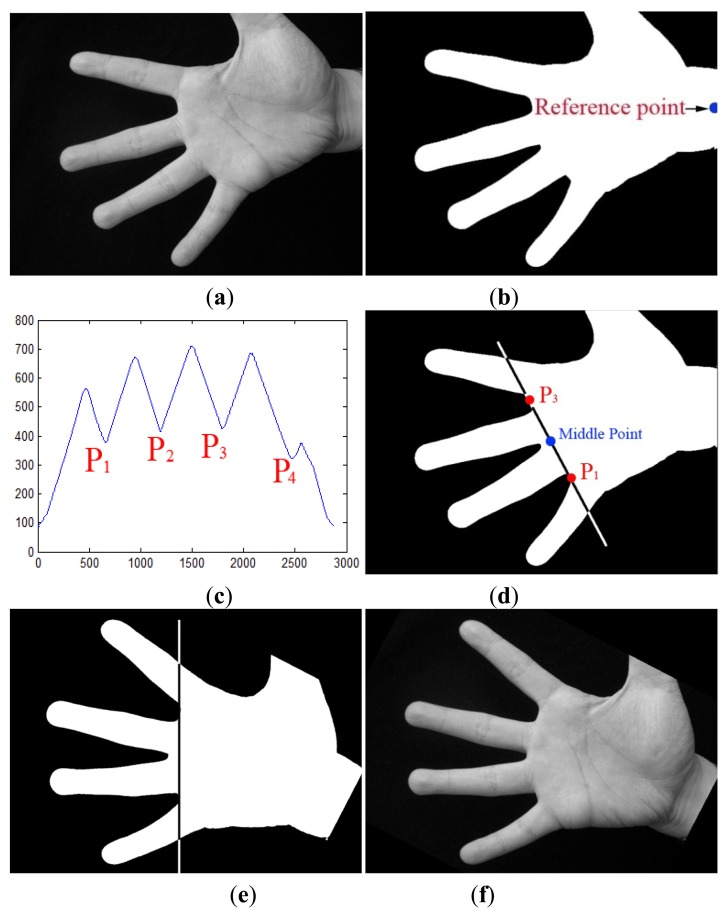
Main steps of the rotation normalization algorithm: (**a**) the gray palmprint image; (**b**) the binary palmprint image and reference point; (**c**) the radial distance function and four key points; (**d**) drawing a line segment P_1_P_3_ can calculate the line's angle; (**e**) rotating the binary image around the middle point of P_1_P_3_ to make it be horizontal; (**f**) the rotation normalized gray palmprint image.

Here, we adopt a classical algorithm to perform rotation normalization [5]. The main steps are described as follows:

**Step 1:** The gray image (see Figure 4(a)) is converted to a binary image (see Figure 4(b)) according to a threshold, which can be obtained by the OTSU algorithm [19].
**Step 2:** One point in the right of binary image located at the center of wrist is selected as the reference point (see Figure 4(b)).
**Step 3:** The radial distance function is calculated. First, the boundary of hand is detected by a boundary tracking algorithm. Next, the distances from the reference point to all boundary points are calculated to get the radial distance function as shown in Figure 4(c). In this function, four minima are detected to obtain four key points (P_1_, P_2_, P_3_, P_4_) corresponding to four gaps between fingers.
**Step 4:** In the binary image, a line segment P_1_P_3_ is drawn between points P_1_ and P_3_ (see Figure 4(d)). Then, the binary image is rotated around the middle point of P_1_P_3_ to make it horizontal. Figure 4f shows the normalized gray image after rotation normalization.

### 2.2. Scale Normalization and ROI Extraction

In the palmprint recognition field, most representative recognition methods are not invariant to scale changes [6]. That is, the training and test samples from a same person should have a same scale. Otherwise, those representative methods would be invalid. As we have mentioned above, scale variance between different images captured from a same palm are inevitable caused by non-contact image acquisition. Thus, scale normalization of palmprint should be done before recognition is performed. In the previous study of non-contact palmprint recognition, some researchers usually performed scale normalization at the vertical direction [14,20]. Here, vertical based scale normalization means that all palmprints should have the same palm width in a certain position of the palms. Han *et al.* [20] proposed a method to estimate the palm width in the center point position.

**Figure 5.**
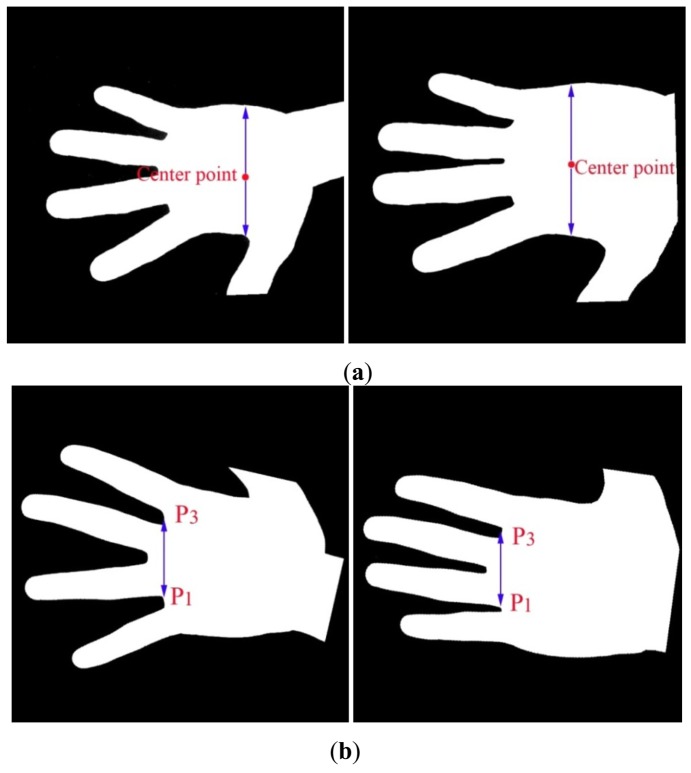
Two existing methods of calculating the palm width or ROI width, (**a**) Han's method to estimate the palm width in the position of center point; (**b**) Michael's method to estimate the size of ROI image using the distance between points P1 and P3.

In Han's method, the gray hand image is converted to a binary image, and then the center point of the binary image is calculated. However, sometimes Han' method cannot accurately calculate the center point position since different hand images may contain different wrist parts. An example is given in Figure 5(a). Michael *et al.* [14] proposed another method to estimate the size of ROI image, in which the distance between points P_1_ and P_3_ is regarded as the width of the ROI image. However, this method is not robust since different hand poses which would lead to changes in the distance between points P_1_ and P_3_, as shown in Figure 5(b).

**Figure 6.**
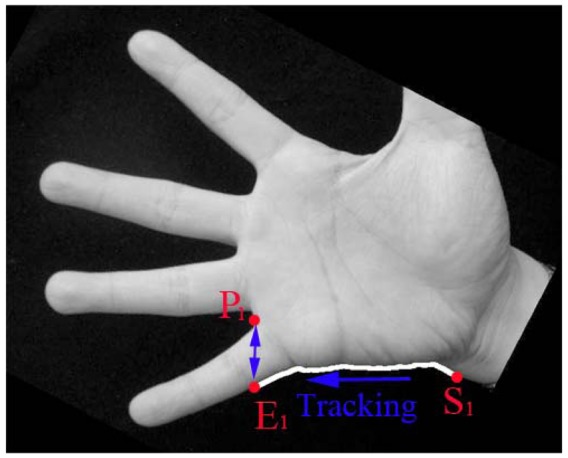
Determine a segment of palm boundary around the start point of heart line by boundary tracking.

**Figure 7.**
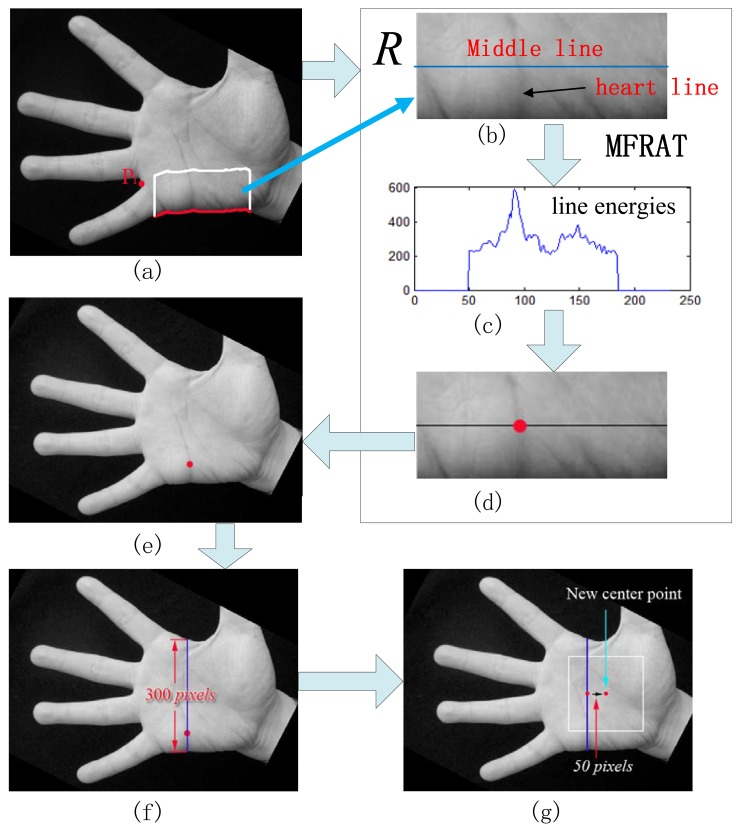
Main steps of the scale normalization algorithm: (**a**) determine a segment of palm boundary around the start point of heart line, and then a rectangle image *R* above the segment is extracted; (**b**) the rectangle image R; (**c**) the line energies across the middle line; (**d**) the detected point (red point) in R; (**e**) the detected point (red point) in the whole palmprint image; (**f**) all palmprint images are resized to have the same height (300 pixels) in the detected point position; (**g**) crop the ROI image.

**Figure 8.**
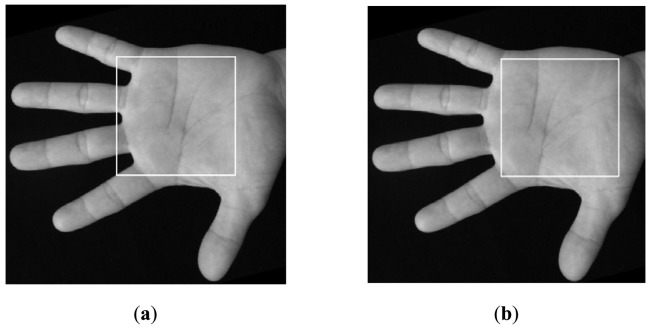
Crop the ROI sub-image before (**a**) and after (**b**) moving the position of center point.

**Figure 9.**
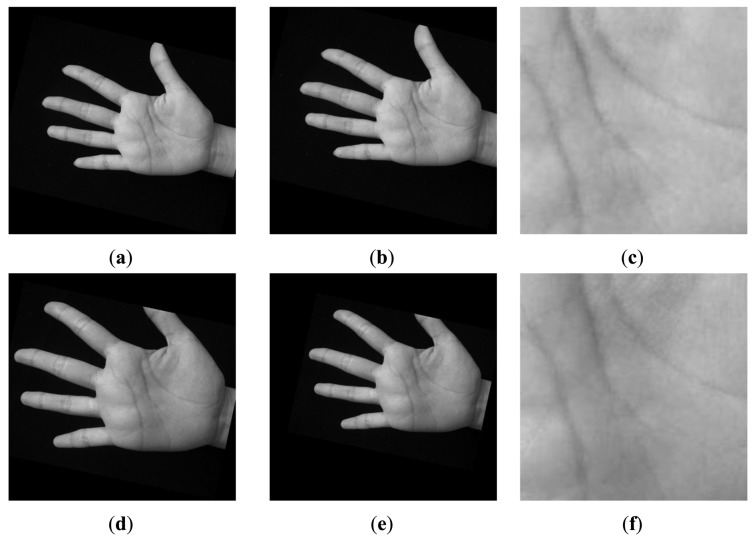
An example of scale normalization: (**a**) and (**d**) two palmprint images with different scales captured from a same palm; (**b**) and (**e**) the scale normalized images of (a) and (d); the ROI images cropped from (b) and (e).

In this paper, we propose an effective algorithm for scale normalization at the vertical direction. As we know, principal lines are the most stable features in palms, and the heart line is near to the point of P_1_ and can be easily detected. Therefore, we try to find a point located in the heart line as the reference position to perform scale normalization. Compared with Han' and Michael's methods, the advantage of our method is that it can calculate the palm width more stably. The main steps of scale normalization and ROI extraction are described as follows:

**Step 1:** Determine a segment of palm boundary around the start point of the heart line. This task can be done using boundary tracking according to the position of P_1_ as shown in Figure 6. In this figure, from the starting point S_1_, we start to track the bottom boundary toward left direction. The tracking will be finished when the last tracking point E_1_ has the same vertical position with P_1_ (see Figure 6). Usually, there is a long distance between the vertical position of P_1_ and head line. We do not need to detect the head line in the area near to the vertical position of P_1_. Therefore, the tracking is stopped in the certain position on the right of P_1_. The distance between this right position and the vertical position of P1 is set to an experiential value in this paper, *i.e.*, 30 pixels, as shown in Figure 7(a). And then, a rectangle image *R* above the segment is extracted (see Figure 7(a)). According our prior knowledge, after rotation normalization, the widths of all palms are between 250 to 400 pixels since the size of whole hand image is 816 × 612 or 778 × 581. Therefore, the height of *R* is set to a suitable value, which is 100 pixels.
**Step 2:** In the image *R*, use modified finite Radon transform (MFRAT) [21,22] of size 100 to calculate the line energies across the middle line as shown Figure 7(b). The detail of MFRAT can be found in literature [21,22]. It can calculate the line energies by comparing the pixels' integration of different lines at the different directions. From Figure 7(b), it can be seen that the point of intersection of middle line of *R* and heart line can be easily detected according to the maximum value of line energies (see Figure 7(c)). The detected point (red point) will used as reference point to perform scale normalization as shown in Figures 7(d,e).
**Step 3:** All palmprint images are resized to have the same height in the detected point position. In this work, the height of normalized palmprint image is 300 pixels as shown in Figure 7(f) since the widths of all palms are between 250 to 400 pixels according our prior knowledge. Here, it should be noted that if the original height of the palm is less than 300 pixels, the width of this palm will be resized to 300 pixels too.
**Step 4:** The middle point of vertical line (blue line) as shown in Figure 7(g) is regarded as the center point of palm. According to this center point, however, the ROI sub-images of some palms cannot be cropped correctly. An example is illustrated in Figure 8(a). In order to better extract ROI sub-image, we move the position of center point toward right direction 50 pixels, which is regarded as the new center point. According to our observation, 50 pixels is a suitable value. If this step is performed, all ROI sub-images can be well cropped in whole database. An example is illustrated in Figure 8(b).
**Step 5:** A square with size of 200 × 200 pixels around the new center point is cropped, which is the ROI image. Finally, we resize the ROI image to a small one, whose size is 128 × 128 pixels. Figure 9 illustrates an example of scale normalization. In this figure, (a) and (d) are two palmprint images captured from a same palm. It can be seen that their scales are obviously different. Figures 9(b) and (e) are their scale normalized images; (c) and (f) are ROI images. From Figure 9, it can be concluded that our scale normalization algorithm is reasonable and effective.

## 3. Recognition Methods

As we have mentioned above, we evaluated the PRADD recognition performances of three different methods, *i.e.*, subspace learning method, correlation method and orientation based method, respectively. Several representative methods are exploited, which will be briefly introduced in the following part of this section.

### 3.1. The Exploited Subspace Learning Methods

Generally, classical subspace learning methods, which are also called appearance methods or subspace analysis methods, seek to find a low-dimensional subspace in a high-dimensional input space by linear transformation. This low-dimensional subspace can provide a compact representation or extract the most discriminant information of the high-dimensional input data. Principal component analysis (PCA) [23] and linear discriminant analysis (LDA) are two typical well known subspace learning methods [23]. PCA is the optimal representation of the input data in the sense of the minimum reconstruction error, which is completely unsupervised because of not taking the class information of the input data into account. In contrast to PCA, LDA takes the class labels into consideration and can produce optimal discriminant projections, which maximizes the ratio of the determinant of the between-class scatter matrix of the projected samples to the determinant of the within-class scatter matrix of the projected samples. It is generally believed that the class information can improve the recognition ability.

In recent years, some important progress has been made in the research on appearance based approaches. Among them, three advances should be highlighted. The first one is the kernel method, which uses a linear classifier algorithm to solve a non-linear problem by mapping the original non-linear observations into a higher-dimensional space [24]. The second one is manifold learning, which is based on the idea that the data points are actually samples from a low-dimensional manifold that is embedded in a high-dimensional space [24]. Manifold learning algorithms aim to uncover the proper parameters in order to find a low-dimensional representation of the data. The last one is matrix and tensor embedding [25–29]. Matrix embedding methods can extract feature matrices using a straightforward image projection [25,26]. Tensor embedding methods represent the image ensembles by a higher-order tensor and extract low-dimensional feature using multilinear algebra methods [27–29]. As we know, kernel PCA (KPCA) and kernel LDA (KLDA) are kernel based versions of PCA and LDA [24], 2DPCA [25] and 2DLDA [26] are matrix based versions of PCA and LDA, and concurrent subspaces analysis (CSA) [27,28] and multilinear discriminant analysis (MDA) [29] are tensor based versions of PCA and LDA. It should be noted that many subspace learning methods have been proposed in recent years. In this paper, due to space limitations, only PCA, LDA and their improved versions mentioned above will be used for PRADD, and Euclidian Distance is used as the similarity measure for these subspace learning methods.

### 3.2. Band-limited Phase-only Correlation Method

Band-Limited Phase-Only Correlation (BLPOC) is an effective and efficient biometrics method proposed for iris recognition by ITO *et al.* [30], which has been successfully applied to palmprint recognition [31]. In this paper, it is also used for PRADD. Firstly, the definition of POC is described as follows: consider two *N*_1_ × *N*_2_ images, *f*(*n_1_, n_2_*), and *g*(*n_1_, n_2_*). Let *F*(*k*_1_,*k*_2_) and *G*(*k*_1_,*k*_2_) denote the 2D Discrete Fourier Transforms (DFTs) of the two images. Here, *F*(*k*_1_,*k*_2_) is given by:

(1) $$\begin{array}{cc} F(k_{1},k_{2}) & =\sum_{n_{1}=\;0\;}^{N_{1}}\sum_{\;n_{2}\;=\;0}^{N_{2}}f(n_{1},n_{2})e^{-j2\pi (\frac{n_{1}k_{1}}{N_{1}}+\frac{n_{2}k_{2}}{N_{2}})}\\ & =A_{F}\;(k_{1},k_{2})\;e^{j\theta_{F}(k_{1},k_{2})} \end{array}$$

where A_F_(*k*_1_,*k*_2_) is amplitude and *θ*_F_(*k*_1_,*k*_2_) is phase. *G*(*k*_1_,*k*_2_) can be defined in the same way. The cross-phase spectrum *R*_FG_(*k*_1_,*k*_2_) is given by:

(2) $$R_{FG}(k_{1},k_{2})=\frac{F(k_{1},k_{2})\bar{G(k_{1},k_{2})}}{|F(k_{1},k_{2})\bar{G(k_{1},k_{2})|}}=e^{j\theta (k_{1},k_{2})}$$

where $\bar{G(k_{1},k_{2})}$ is the complex conjugate of *G*(*k*_1_,*k*_2_) and *θ*(*k*_1_,*k*_2_) denotes the phase difference *θ*_F_(*k*_1_,*k*_2_)-*θ*_G_(*k*_1_,*k*_2_). The POC function *r_fg_*(*n*_1_,*n*_2_) is the 2D Inverse DFT (2D IDFT) of *R*_FG_(*k*_1_,*k*_2_) and is given by:

(3) $$r_{fg}(n_{1},n_{2})=\frac{1}{N_{1}N_{2}}\sum_{k_{1}k_{2}}e^{j\theta (k_{1},k_{2})}e^{j2\pi (\frac{n_{1}k_{1}}{N_{1}}+\frac{n_{2}k_{2}}{N_{2}})}$$

From Equations (2) and (3), we can see that original POC exploits all components of the image's 2D DFT to generate the out plane. In [30], ITO *et al.* found that BLPOC can achieve better recognition performance by removing the high frequency components and only using the inherent frequency band for matching.

Here we denote the center area of *θ*_F_(*k*_1_,*k*_2_) and *θ*_G_(*k*_1_,*k*_2_) as *θ*_F_(*k*_1_,*k*_2_)_BL_ and *θ*_G_(*k*_1_,*k*_2_)_BL_, whose size is *J*_1_ × *J*_2_. Thus, the BLPOC function is given by:

(4) $$r_{fg}{\left(n_{1},n_{2}\right)}_{BL}=\frac{1}{J_{1}J_{2}}\sum_{k_{1}k_{2}}e^{j(\theta_{F}{\left(k_{1},k_{2}\right)}_{BL}-\theta_{G}{\left(k_{1},k_{2}\right)}_{BL})}e^{j2\pi (\frac{n_{1}k_{1}}{J_{1}}+\frac{n_{2}k_{2}}{J_{2}})}$$

Finally, the 1D vector, *r_fg_*(*n*_1_,*n*_2_)_BL_, should be converted to 2D array by lexicographic ordering to generate the correlation output plane (COP).

For correlation based methods, three values, *i.e.*, *peak*, *peak-to-correlation energy* (*PCE*), and *peak-to-sidelobe ratio* (*PSR*) were often adopted as similarity measures [32,33]. As the name suggests, *peak* is the maximum peak value in COP. *PCE* and *PSR* are defined by:

(5) $$\text{PCE}=\frac{\text{peak}-\text{mea}n_{\text{COP}}}{std_{\text{COP}}},\;\text{PSR}=\frac{\text{peak}-\text{mea}n_{\text{sidelobe}}}{std_{\text{sidelobe}}}$$

where *mean_COP_* is the average of the COP, *std_COP_* is the standard deviation of the COP, *mean_sidelobe_* is the average of the sidelobe region surrounding the peak (21 × 21 pixels with a 5 × 5 excluded zone around the peak), and *std_sidelobe_* is the standard deviation of the sidelobe region values. In our previous work [34], we found *PSR* is a better measure than peak and *PCE*. Thus, we select *PSR* as the similarity measure in this paper.

### 3.3. The Exploited Orientation Coding Based Methods

In this paper, three classical orientation coding based methods are used for PRADD, which are Ordinal Code [12], Competitive Code (CompCode) [35], and Robust Line Orientation Code (RLOC) [22], respectively.

#### 3.3.1. Ordinal Code

In Ordinal Code [12], 2D Gaussian filter is exploited for extracting the line energy of every pixel in a palmprint. The form of 2D Gaussian filter is given as follows:

(6) $$f(x,y,\theta )=\exp[-{\left(\frac{x\cos\theta +y\sin\theta }{\delta_{x}}\right)}^{2}-{\left(\frac{-x\sin\theta +y\cos\theta }{\delta_{y}}\right)}^{2}]$$

where *θ* denotes the orientation of 2D Gaussian filter, *δ_x_* and *δ_y_* denote the filter's horizontal scale and vertical scale, respectively. And then the orthogonal line ordinal filter (*OF*) can be designed as follows:

(7) $$OF(\theta )=f(x,y,\theta )-f\left(x,y,\theta +\frac{\pi }{2}\right)$$

In [12], three ordinal filters, *OF*(0), *OF*(*π*/6) and *OF*(*π*/3), were exploited to extract the ordinal feature. The main steps of feature extraction are presented as follows:

**Step 1:** Using *OF*(0) to filter a preprocessed palmprint image *I*(*x, y*) to get filtered image *OF*(0)_image:
  (8) OF(0)_image=I(x,y)∗OF(0)
  where * means convolution processing.
**Step 2:** The Ordinal Code (obtained from *OF*(0)_image) can be gotten according to the sign of filtering results:
  (9) $$\text{Ordinal Code}\;{\left(x,y\right)}_{0}=\{\begin{array}{c} 1,\;\text{if}\;OF(0)\_\text{image}\;(x,y)>0\\ 0,\;\text{if}\;OF(0)\_\text{image}\;(x,y)<0 \end{array}$$
**Step 3:** Repeat Step 1 and 2 using filters *OF*(*π*/6) and *OF*(*π*/3). As a result, we get three bit plane of Ordinal Code.

In matching stage, Hamming distance is exploited for the similarity measure. If *A* is the feature of a training sample with the size of *M* × *N*, and *B* is the feature of a test sample with the same size, the Hamming distance (*D*(*A*,*B*)) between them is defined as follows:

(10) $$D(A,B)=\frac{\sum_{x=1}^{M}\sum_{y=1}^{N}\sum_{i}^{3}\left(A_{i}^{b}(x,y)⊗B_{i}^{b}(x,y)\right)}{3\times M\times N}$$

where ⊗ is bitwise exclusive OR and $A_{i}^{b}$ (or $B_{i}^{b}$) is the *i*th bit plane of *A* (or *B*).

Theoretically speaking, (*D*(*A,B*))is between 0 and 1, and the smaller the matching score the greater the similarity between *A* and *B*. The matching score of a perfect match is 0.

#### 3.3.2. CompCode

The basic idea of Competitive Code is to extract the orientation field as features by 2D ellipsoidal Gabor filter bank and use angular distance as a matching function [35]. Generally speaking, 2D ellipsoidal Gabor filter has the following form:

(11) $$\psi (x,y,\omega ,\theta )=\frac{\omega }{\sqrt{2\pi k}}{\text{e}}^{-\frac{\omega^{2}}{8k^{2}}(4x^{\prime 2}+y^{\prime 2})}\left({\text{e}}^{i\omega x\prime }-{\text{e}}^{-\frac{k^{2}}{2}}\right)$$

where *x*′ = (*x* – *x*_0_)cosθ + (*y* – *y*_0_)sinθ, y′ = –(*x* – *x*_0_)sinθ + (*y* – *y*_0_)cosθ is the center of the function. ω is the radial frequency in radians per unit length and *θ* is the orientation of the Gabor functions in radians. *k* is defined as $k=\sqrt{2\ln2}\left(\frac{2^{\delta }+1}{2^{\delta }-1}\right)$, where *δ* is the half-amplitude bandwidth of the frequency response. Based on this Gabor function, a Gabor filter bank with one scale and six directions are created:

(12) $$\begin{array}{ll} \theta_{k}=\frac{\pi (k-1)}{6},\; & k=1,2,\ldots ,6. \end{array}$$

A brief summary of Competitive Code is given below:

**Step 1:** Six real parts of Gabor filters *ψ*(*x, y, ω, θ_k_*) with different directions *θ_k_* are applied to a preprocessed palmprint image *I* (*x, y*).
**Step 2:** The orientation of a local region is obtained by the competitive rule
  (13) $$k=\arg\;min_{k}(I(x,y)*\psi (x,y,\omega ,\theta_{k}))(k=\{1,2,3,4,5,6\})$$

Two Competitive Codes are compared by their angular distance. The implementation of calculating angular distance is also based on Hamming distance.

#### 3.3.3. Robust Line Orientation Code

RLOC is another effective orientation based approach, which use the MFRAT to extract the orientation feature [21,22]. The MFRAT and RLOC are introduced as follows: denoting Z*_p_* = {0, 1,…, *p* – 1}, where *p* is a positive integer, the MFRAT of real function *f*[*x*,*y*] on the finite grid *Z_p_^2^* is defined as:

(14) $$r[L_{k}]=\text{MFRA}T_{f}(k)=\sum_{i,j\in L_{k}}f[i,j]$$

where *L_k_* denotes the set of points that make up a line on the lattice *Z_p_^2^*, which means:

(15) $$L_{k}=\{(i,j):j=S_{k}\;(i-i_{0})+j_{0},i\in Z_{p}\}$$

where (*i*_0_,*j*_0_) denotes the center point of the lattice *Z_p_^2^*, and *k* means the index value corresponding to a slope of *S_k_*. That is to say, different *k* denotes different slopes of *L_k_*. For any given *k*, the summation *r*[*L_k_*] of only one line, which passes through the center point (*i*_0_,*j*_0_) of *Z_p_^2^*, is calculated. Actually, *r*[*L_k_*] is the energy of line *L_k_*. In order to make a correct energy comparison among all lines, lines at different directions should have an identical number of pixels. The discussions about the differences between finite radon transform (FRAT) and MFRAT can be found in [21].

In the MFRAT, if there exist a genuine line which passes through the center point (*i*_0_,*j*_0_) of *Z_p_^2^*, we can obtain its index value of direction *k*_min_(*i*_0_,*j*_0_) by the following formula:

(16) $$\begin{array}{ll} k_{\min}\;(i_{0},j_{0})=\arg\underset{k}{\min}(r[L_{k}]) & k=1,2,\cdots N \end{array}$$

In this way, the directions of all pixels can be calculated if the center of lattice *Z^2^_p_* moves over an image pixel by pixel. In RLOC, the pixel-to-area distance was exploited for matching. The matching score from *A* to *B* is defined as follows:

(17) $$s(A,B)=\left(\sum_{i=1}^{m}\sum_{j=1}^{n}A(i,j)\cup \bar{B}(i,j)\right)/m\times n$$

where “U” is the logical “EQUAL” operation, which means that the value of *A*(*i, j*) ∪ *B*(*i, j*) will be 1 if *A*(*i, j*) and at least one point of *B̄* (*i, j*) are equal, otherwise it will be 0. And *B̄* (*i, j*) is defined as cross-shaped area around *B*(*i, j*), which is (*B*(*i* − 1,*j*), *B*(*i +* 1, *j*), *B*(*i*, *j*), *B*(*i*,*j* − 1), *B*(*i*,*j +* 1)).

In a similar way, the matching score from *B* to *A* can also be defined as:

(18) $$s(B,A)=\left(\sum_{i=1}^{m}\sum_{j=1}^{n}B(i,j)\cup \bar{A}(i,j)\right)/m\times n$$

Finally, the matching score between *A* and *B* is defined as:

(19) S(A,B)=S(B,A)=Max(s(A,B),s(B,A))

Theoretically speaking, *S*(*A*,*B*) is between 0 and 1, and the larger the matching score the greater the similarity between *A* and *B*. The matching score of a perfect match is 1.

## 4. Experiments

In this section, intra-sensor and inter-sensor experiments are conducted, respectively. In intra-sensor experiments, the training set and the test set are captured from a same device. Obviously, in inter-sensor experiments the training set and the test set are captured from different devices. In this paper, the nearest neighbor rule (1NN) is used for classification.

### 4.1. Experimental Results of Intra-Sensor Recognition

We firstly conduct the intra-sensor experiments on three sub-databases. In these three sub-databases, the 1∼3, 4∼6, and 7∼9 samples captured in the first session are used for training, respectively, and the 10 samples from the second session are used for test. That is, for each method, the experiment will be conducted three times in one sub-database.

In the first experiment, we test the recognition performance of subspace learning methods. Only the identification experiments are conducted. Identification is a one-to-many comparison against all stored templates, which answers the question of “who is this person”? In the identification experiments, the statistical value of Best Identification Rate (BIR) is adopted to evaluate the performances of different methods. For a subspace learning method, given a number of dimension we can obtain one identification rate. When the number of dimension varies, many identification rates can be obtained. At last, the highest identification rate will be regarded as the BIR. The BIRs and corresponding dimensions of different subspace learning methods conducted three times are listed in Table 1. From this Table, it can be seen that the BIRs of subspace learning methods are not satisfactory since the highest BIR of all methods is only about 90%. The average BIR of different subspace learning methods for intra-sensor recognition are listed in the last row using bold fonts in Table 1.

In the second experiment, we test the recognition performances of correlation based and orientation based methods, *i.e.*, BLPOC, CompCode, Ordinal Code and RLOC, respectively. It should be noted that both identification and verification experiments are conducted. Generally, verification is a one-to-one comparison against a single stored template, which answers the question of “whether the person is whom he claims to be”. In the verification experiments, the statistical value of Equal Error Rate (EER) is adopted to evaluate the performance of different methods.

In the experiments of BLPOC, determining suitable values of *J*_1_ and *J*_2_ is a key problem that should be solved firstly. Since the ROI image of palmprint is a square, and its Fourier spectrum is also a square, we let *J*_1_ equal to *J*_2_. That is to say, the selected center area of the 2D DFT spectrum is also a square, whose size is *J*_1_ × *J*_1_. Furthermore, in order to choose the best *J*_1_, we conduct the tests exploiting different values of *J*_1_. Here, the values of *J*_1_ are set to an even number, and the range of *J*_1_ is {22, 24, (x022EF), 40}.

In Ordinal Code, two parameters of 2D Gaussian filter, *δ_x_* and *δ_y_*, were set to 5 and 1, respectively. In Competitive Code, two parameters of 2D ellipsoidal Gabor filter, *ω* and *δ*, were set to 0.5 and 1.5, respectively. Meanwhile, the size of all filters mentioned above is 40 × 40. In RLOC, we use 16 × 16 MFRAT, whose width of the lines, *L_k_*, is 4 pixels, to extract RLOC feature [22].

The BIRs and EERs of the BLPOC, CompCode, Ordinal Code, and RLOC methods on three sub-databases are listed in Table 2. The average BIR or EER of these methods for intra-sensor recognition are also listed in the last row using bold fonts in Table 2. From this table, it can be seen that the orientation coding based methods achieve satisfying BIRs, which are near 100%, and the recognition performance of BLPOC is obviously worse than that of orientation coding based methods. Meanwhile, it can be observed that the BIRs and EERs of orientation coding based methods obtained from sub-database C950 is a little better than that of N5800 and M525. It should be noted that the recognition performances of CompCode and Ordinal Code is stable on three sub-databases while the recognition performance of RLOC is easily influenced by image quality.

**Figure 10.**
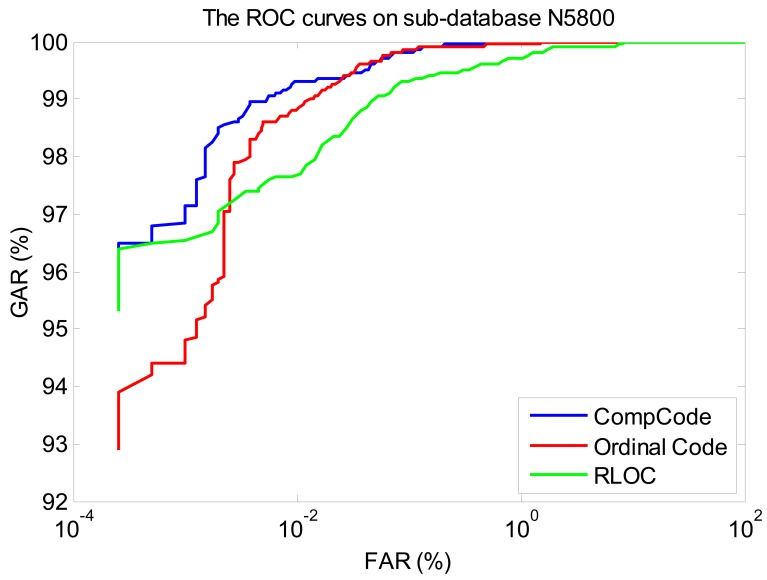
The ROC curves of methods Comp Code, Ordinal Code and RLOC on sub-database N5800 (using the 1∼3 samples of the first session as the training set).

**Figure 11.**
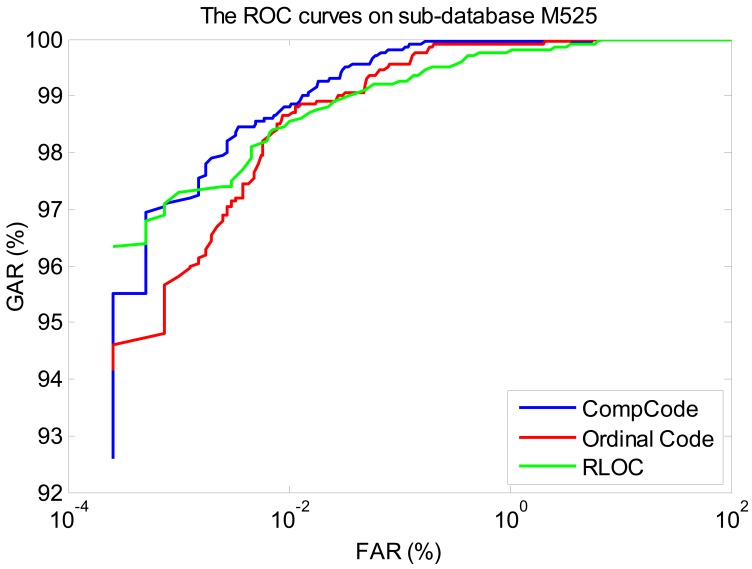
The ROC curves of methods Comp Code, Ordinal Code and RLOC on sub-database M525 (using the 1∼3 samples of the first session as the training set).

**Figure 12.**
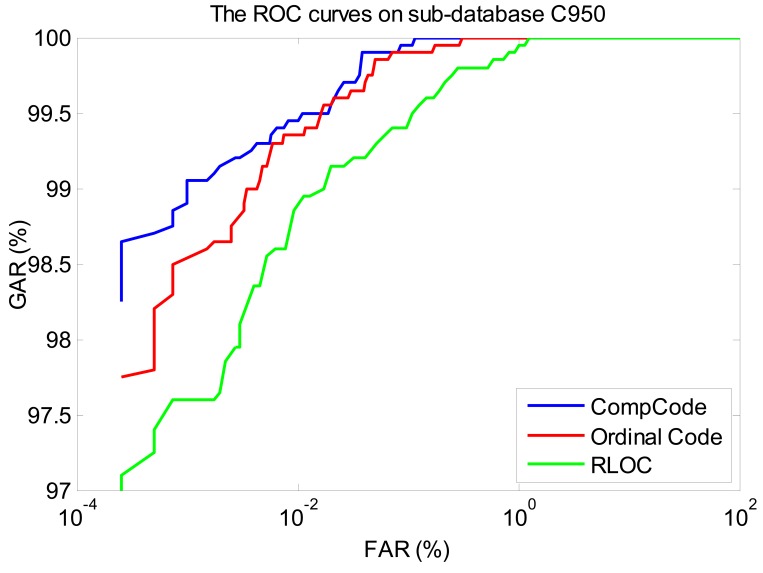
The ROC curves of methods Comp Code, Ordinal Code and RLOC on sub-database M525 (using the 1∼3 samples of the first session as the training set).

In order to better illustrate the recognition performances of three orientation coding based methods on three sub-databases, their Receiver Operating Characteristic (ROC) curves (the experiments using the 1∼3 samples of the first session as the training set) are illustrated in Figures 10–12, which plot the False Accept Rate (FAR) against the Genuine Accept Rate (GAR).

### 4.2. Experimental Results of PRADD

In this section, we evaluate the recognition performances of different methods for PRADD (inter-sensors recognition), therefore, the experiments are designed across different sub-databases. To do so, we construct three new across-sub-databases. In the first across-sub-database, the 1∼3, 4∼6, and 7∼9 samples from the first session in sub-database N5800 are used for training, respectively, and the 20 samples from the second session in sub-databases M525 and C950 are used for testing. That is to say, the experiment will be conducted three times using different training sets. For convenience, this new across-sub-database is named as A_N5800. Consequently, in A_N5800 the numbers of samples for training and test are 1800 and 4000, respectively. In the similar way, two other across-sub-databases, *i.e.*, A_M525, A_C950, are constructed. The details of three across-sub-databases, A_N5800, A_M525 and A_C 950 are listed in Table 3.

The BIRs and corresponding dimensions of different subspace learning methods obtained from three times experiments for PRADD are listed in Table 4. The average BIR of different subspace learning methods for inter-sensor recognition are also listed in the last row using bold fonts in Table 4. Compared with Table 1, it can be seen from Table 4 that the BIRs of subspace learning methods are poor for inter-sensor recognition. Therefore, it can be concluded that subspace learning methods are not suitable for PRADD.

The BIRs and EERs of the BLPOC, Comp Code, Ordinal Code, and RLOC methods on three across-sub-databases are listed in Table 5. Compared with Table 2, it can be seen that the recognition performances of these methods decrease a little. In these four methods, the orientation coding based methods CompCode and Ordinal Code also achieve promising BIRs and EER_S_. In other words, the recognition performances of CompCode and Ordinal Code are rather stable.

In Table 5, in the first and second experiment, an interesting phenomenon is that the recognition performances of four methods on across-sub-database A_C950 are obviously worse than that of on A_N5800 and A_M525. However, in the third experiment, the situation becomes reversed. This phenomenon may be caused by the preprocessing method or the hand poses during image acquisition. For example, several samples within the first six samples in the C950 database may be not well cropped, or they have obvious affine transformations caused by different hand poses. Thus, they cannot well match with test samples captured by smart phones.

**Figure 13.**
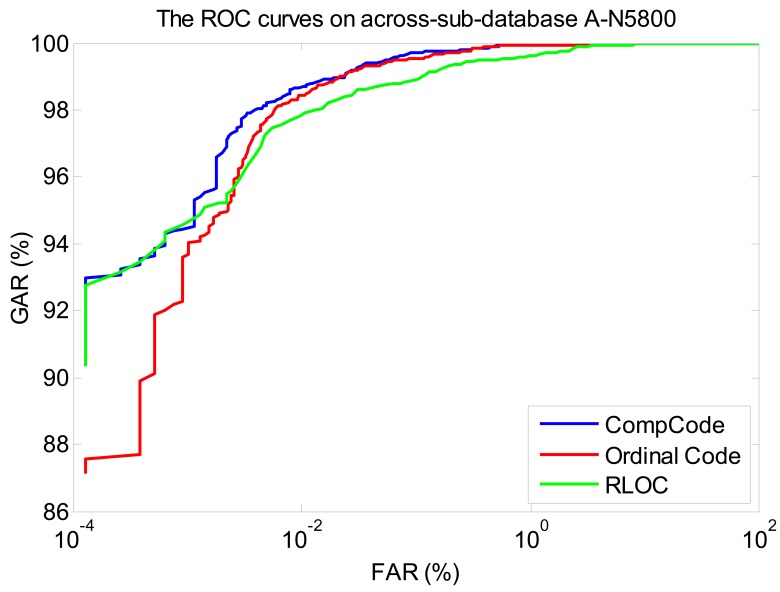
The ROC curves of methods Comp Code, Ordinal Code and RLOC on across-sub-database A_N5800 (using the 1∼3 samples of the first session as the training set).

**Figure 14.**
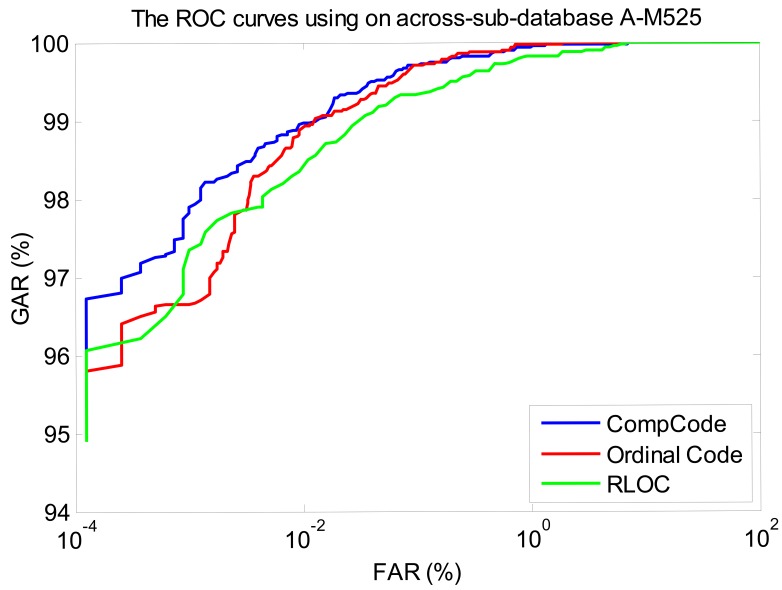
The ROC curves of methods Comp Code, Ordinal Code and RLOC on across-sub-database A_M525 (using the 1∼3 samples of the first session as the training set).

**Figure 15.**
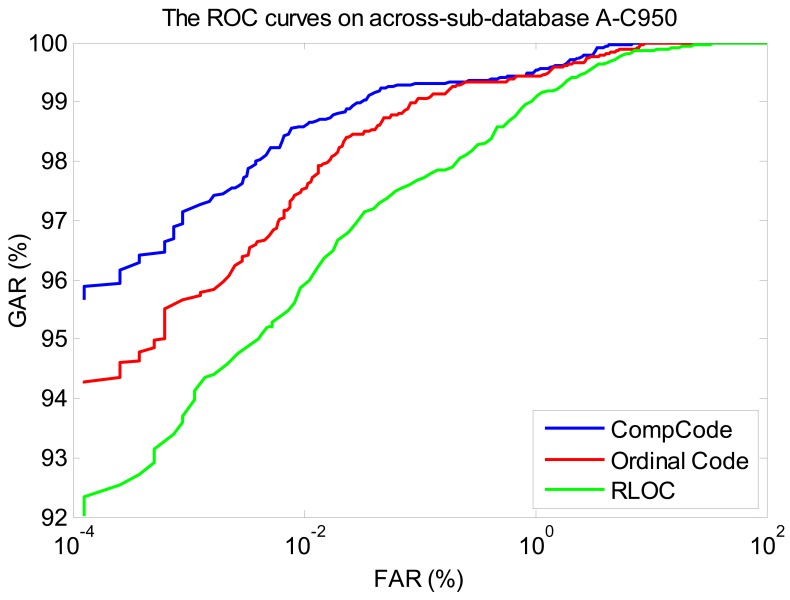
The ROC curves of methods Comp Code, Ordinal Code and RLOC on across-sub-database A_C950 (using the 1∼3 samples of the first session as the training set).

We also illustrate the ROC curves (the curves of the experiments using the 1∼3 samples of the first session as the training set) of three orientation coding based methods on three sub-databases, in Figures 13–15, in which it can be seen that the method of CompCode also achieves the best recognition performance for PRADD.

### 4.3. Performance Comparisons between Intra-Sensor and Inter-Sensor Recognition

In this section, the performance comparisons between intra-sensor and inter-sensor recognition are illustrated.

**Figure 16.**
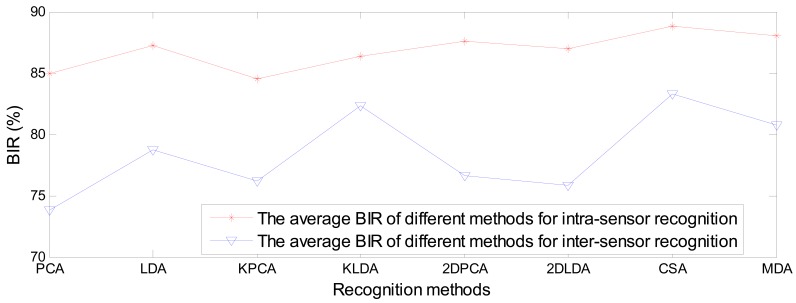
The average BIR comparisons of different subspace learning methods between intra-sensor recognition and inter-sensor recognition.

Figure 16 shows the average BIR comparisons of different subspace learning methods between intra-sensor recognition and inter-sensor recognition. The average BIR of different subspace leaning methods for intra-sensor recognition is in the range 85%∼89%, while the BIR for inter-sensor recognition is in the range 74%∼83%, which are poor recognition performance.

**Figure 17.**
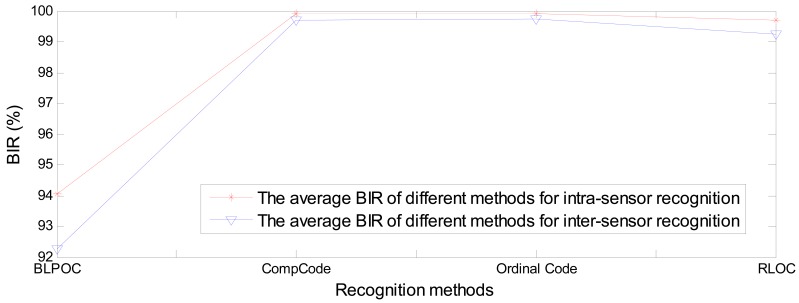
The average BIR comparisons of BLPOC method and three orientation coding based methods between intra-sensor recognition and inter-sensor recognition.

**Figure 18.**
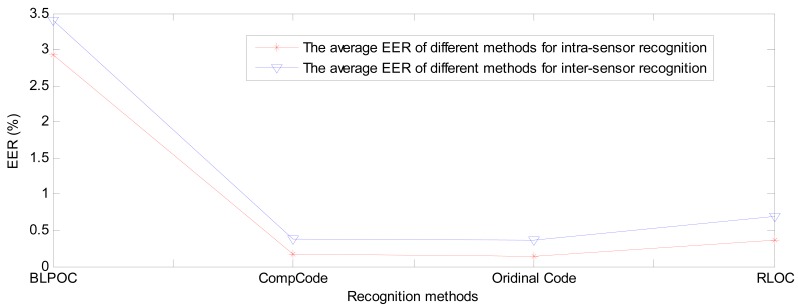
The average EER comparisons of BLPOC method and three orientation coding based methods between intra-sensor recognition and inter-sensor recognition.

Figures 17 and 18 show the average BIR and EER comparisons of BLPOC method and three orientation coding based methods between intra-sensor recognition and inter-sensor recognition. It can be seen that the performance of orientation coding based methods are far better than that of BLPOC.

Among the three orientation coding based methods, the performances of CompCode and Ordinal Code are very close and slightly better than that of RLOC. The average BIR and EER of CompCode and Oridinal Code for intra-sensor recognition are 99.91% and 0.168% and 99.91% and 0.136%, respectively, while the BIR and EER of them for inter-sensor recognition are 99.71% and 0.375% and 99.73% and 0.371%, respectively. The reasons why the CompCode and Ordinal Code are rather stable in terms of the performance are twofold. First, the orientation feature is inherently robust to noise, illumination changes and contrast changes, which has been partly proven in previous studies [12,22,35]. Second, in CompCode and Ordinal Code, the sizes of 2D ellipsoidal Gabor filter and 2D Gaussian filter are all 40 × 40, which is large enough for robust recognition. From Figure 3, it can be seen that the quality of image captured by the C950 is very good, and it seems that the quality of images captured by M525 and N5800 is obviously worse than that of C950. In the image captured by C950, all palm lines including principal lines, large wrinkles and small wrinkles are very clear, as shown in Figure 3(a). In the images captured by M525 and N5800, the principal lines and large wrinkles are clear, but small wrinkles are unclear. Due to a large filter size, CompCode and Ordinal Code can extract robust features located in principal lines and large wrinkles from images captured by different devices, and the features located in small wrinkles will be neglected. On the contrary, in RLOC, MFRAT with size of 16 × 16 is used for feature extraction, which is sensitive to changes of small wrinkles. From above analysis, it is not strange that why CompCode and Ordinal Code achieve good performance for PRADD while RLOC cannot.

## 5. Conclusions

In this paper, we investigated the problem of Palmprint Recognition Across Different Devices (PRADD). It should be noted that it is the first time this problem has been studied in the palmprint recognition field. In order to conduct this research, we created a PRADD image database containing 12,000 grayscale captured from 100 subjects using three devices, *i.e.*, one digital camera and two smart-phones. Using this database, we evaluate the recognition performances of three different methods, *i.e.*, subspace learning method, correlation method and orientation coding based method, respectively. According to experiments results, several meaningful conclusions can be obtained: (1) Three popular consumer electronics products including one digital camera and two smart-phones were used to create palmprint image databases, and good recognition performance was obtained on these databases. Therefore, it can be concluded that these consumer electronics products are suitable for use in the technique of palmprint recognition. (2) The proposed scale normalization algorithm for PRADD is reasonable and effective. (3) On three across-sub-databases, orientation coding based methods, especially CompCode and Ordinal Code achieve promising recognition performance for PRADD. That is to say, these two methods are suitable for PRADD. (4) Since promising recognition performances are obtained for PRADD, it can be concluded that palmprints are a good human trait, which can be used across different capture devices. In our future work, we will try to exploit other strategies to further improve the recognition performance of PRADD. For example, we will develop multi-feature based methods to achieve better performance for PRADD.

## Figures and Tables

**Table 1.** BIRs (%) and corresponding dimensions (the number in the bracket) of different subspace learning methods conducted three times on three sub-databases.

	**PCA**	**LDA**	**KPCA**	**KLDA**	**2DPCA**	**2DLDA**	**CSA**	**MDA**
N5800	85.75 (250)	89.75 (80)	86.85 (220)	88.05 (170)	87.30 (17 × 128)	87.45 (11 × 128)	89.95 (12 × 12)	88.65 (10 × 10)
	85.50 (200)	89.85 (70)	87.15 (230)	86.80 (190)	87.10 (14 × 128)	86.00 (10 × 128)	90.03 (11 × 11)	89.25 (13 × 13)
	86.00 (210)	88.00 (100)	86.15 (250)	86.75 (190)	88.00 (17 × 128)	87.95 (11 × 128)	88.95 (11 × 128)	87.85 (12 × 128)
M525	83.90 (200)	85.95 (80)	85.70 (230)	87.75 (190)	86.60 (11 × 128)	86.95 (10 × 128)	87.95 (13 × 13)	87.40 (11 × 11)
	85.35 (240)	88.10 (100)	85.80 (230)	85.80 (180)	86.85 (15 × 128)	87.85 (12 × 128)	88.55 (12 × 12)	88.35 (10 × 10)
	82.50 (230)	83.50 (80)	82.85 (240)	83.85 (190)	86.30 (13 × 128)	85.15 (13 × 128)	84.60 (13 × 13)	85.30 (12 × 12)
C950	84.85 (230)	88.05 (100)	81.50 (250)	86.10 (190)	88.45 (14 × 128)	88.70 (15 × 128)	90.30 (13 × 13)	89.85 (12 × 12)
	84.25 (300)	85.35 (80)	82.15 (250)	87.50 (190)	87.95 (18 × 128)	85.75 (15 × 128)	89.20 (12 × 12)	87.35 (13 × 13)
	86.35 (300)	86.75 (100)	82.55 (250)	84.90 (170)	89.55 (17 × 128)	87.05 (12 × 128)	90.05 (12 × 12)	88.15 (12 × 12)
**Average BIR**	**84.94**	**87.25**	**84.5**	**86.4**	**87.57**	**86.98**	**88.84**	**88.01**

**Table 2.** BIRs and EERs of BLPOC and three orientation coding based methods conducted three times on three sub-databases (The value of *J_1_* in BLPOC method achieved highest BIR or lowest EER is listed in brackets).

	**BIR (%)**				**EER (%)**			

	**BLPOC**	**Comp Code**	**Ordinal Code**	**RLOC**	**BLPOC**	**Comp Code**	**Ordinal Code**	**RLOC**
N5800	93.800 (22)	99.950	100	99.60	2.919 (22)	0.117	0.111	0.420
	93.050 (26)	99.950	99.950	99.700	3.050 (26)	0.220	0.120	0.240
	92.300 (28)	99.750	99.750	99.650	3.690 (26)	0.270	0.220	0.630
M525	94.900 (26)	99.950	99.900	99.700	2.699 (22)	0.112	0.168	0.360
	94.700 (26)	99.950	99.900	99.600	2.500 (26)	0.100	0.060	0.400
	92.500 (22)	99.850	99.950	99.550	2.900 (24)	0.220	0.220	0.550
C950	95.300 (28)	99.950	99.950	99.900	2.820 (28)	0.068	0.080	0.250
	95.000 (30)	99.900	99.900	99.800	2.940 (30)	0.125	0.170	0.190
	94.950 (28)	99.950	99.900	99.800	2.790 (28)	0.280	0.080	0.200
**Average BIR,EER**	**94.050**	**99.910**	**99.910**	**99.700**	**2.920**	**0.168**	**0.136**	**0.360**

**Table 3.** The details of three across-sub-databases, A_N5800, A_M525 and A_C 950.

	**Training Set**	**Test Set**
A_N5800	The 1∼3, 4∼6, and 7∼9 samples from the first session in sub-database N5800 are used for training, respectively.	the 20 samples from the second session in sub-databases M525 and C950
A_M525	The 1∼3, 4∼6, and 7∼9 samples from the first session in sub-database M525 are used for training, respectively.	the 20 samples from the second session in sub-databases N5800 and C950
A_C950	The 1∼3, 4∼6, and 7∼9 samples from the first session in sub-database C950 are used for training, respectively.	the 20 samples from the second session in sub-databases M525 and N5800
*Total number*	1,800	4,000

**Table 4.** BIRs (%) and corresponding dimensions (the number in the bracket) of different subspace learning methods conducted three times on three across-sub-databases.

	**PCA**	**LDA**	**KPCA**	**KLDA**	**2DPCA**	**2DLDA**	**CSA**	**MDA**
A_N5800	70.40 (210)	77.48 (110)	75.22 (220)	84.15 (190)	74.90 (14 × 128)	73.70 (9 × 128)	83.37 (12 × 12)	79.20 (11 × 11)
	72.25 (290)	77.08 (110)	77.22 (210)	83.15 (190)	78.10 (18 × 128)	67.17 (10 × 128)	83.40 (12 × 12)	77.02 (14 × 14)
	71.83 (240)	76.58 (100)	76 (220)	80.95 (190)	76.10 (18 × 128)	73.82 (10 × 128)	81.60 (13 × 13)	76.15 (9 × 9)
A_M525	81.93 (270)	89.28 (90)	84.55 (250)	82.20 (190)	81.03 (17 × 128)	81.70 (8 × 128)	89.65 (12 × 12)	86.95 (9 × 9)
	82.15 (280)	90.33 (110)	84.55 (300)	83.45 (190)	80.57 (17 × 128)	81.03 (8 × 128)	90.33 (13 × 13)	88.48 (9 × 9)
	81.05 (300)	88.28 (100)	80.73 (320)	82.15 (190)	77.32 (18 × 128)	77.42 (7 × 128)	88.45 (13 × 13)	84.40 (13 × 13)
A_C950	68.87 (210)	71.60 (170)	71.03 (220)	80.13 (190)	74.20 (12 × 128)	76.50 (12 × 128)	79.30 (12 × 12)	78.73 (12 × 12)
	64.98 (210)	65.25 (110)	65.15 (230)	84.55 (190)	71.60 (18 × 128)	75.85 (14 × 128)	75.25 (13 × 13)	76.02 (12 × 12)
	70.57 (250)	72.65 (200)	71.00 (220)	80.17 (140)	75.45 (17 × 128)	74.98 (14 × 128)	78.00 (12 × 12)	79.47 (12 × 12)
**Average BIR**	**73.78**	**78.73**	**76.16**	**82.32**	**76.58**	**75.8**	**83.26**	**80.71**

**Table 5.** BIRs and EERs of BLPOC and three orientation coding based methods conducted three times on three across-sub-databases (The value of *J_1_* in BLPOC method achieved highest BIR or lowest EER is listed in brackets).

	**BIR (%)**				**EER (%)**			

	**BLPOC**	**Comp Code**	**Ordinal Code**	**RLOC**	**BLPOC**	**Comp Code**	**Ordinal Code**	**RLOC**
A_N5800	93.070 (22)	99.925	99.950	99.550	3.345 (22)	0.220	0.250	0.500
	92.570 (22)	99.850	99.850	99.550	3.270 (24)	0.270	0.220	0.500
	91.350 (24)	99.500	99.425	98.875	3.650 (22)	0.440	0.390	0.875
A_M525	93.225 (26)	99.950	99.900	99.725	3.024 (28)	0.200	0.190	0.350
	91.750 (24)	99.825	99.825	99.425	3.070 (28)	0.270	0.300	0.520
	90.975 (22)	99.450	99.550	98.970	3.470 (28)	0.520	0.510	0.900
A_C950	92.470 (24)	99.625	99.570	99.000	3.848 (28)	0.580	0.600	0.960
	92.550 (26)	99.525	99.600	98.925	3.650 (28)	0.480	0.550	0.970
	92.500 (26)	99.800	99.925	99.225	3.300 (28)	0.400	0.330	0.600
**Average BIR,EER**	**92.270**	**99.710**	**99.730**	**99.240**	**3.400**	**0.375**	**0.371**	**0.686**
